# Blood gas analysis: Clinical applications, interpretation and future directions (Review)

**DOI:** 10.3892/mi.2025.291

**Published:** 2025-12-16

**Authors:** Mercedes Núñez Sanagustín, Joško Osredkar

**Affiliations:** 1 Institute of Clinical Chemistry and Biochemistry, University Medical Centre Ljubljana, 1000 Ljubljana, Slovenia; 2 Faculty of Pharmacy, University of Ljubljana, 1000 Ljubljana, Slovenia

**Keywords:** arterial blood gas (ABG), acid-base disorders, respiratory failure, clinical diagnostics, artificial intelligence in medicine

## Abstract

Blood gas analysis represents a cornerstone diagnostic method in clinical practice, providing rapid assessment of respiratory and metabolic status through evaluation of pH, partial pressure of oxygen, partial pressure of carbon dioxide and bicarbonate. The present comprehensive review discusses recent advances in blood gas analysis, including emerging artificial intelligence (AI) applications, controversial practices in venous vs. arterial sampling and closed-loop management systems in critical care. The present review critically synthesizes evidence from recent systematic reviews and meta-analyses, addressing key controversies, such as the clinical utility of venous blood gas analysis with venous-to-arterial conversion technology (sensitivity, 97.6%; specificity, 36.9% for respiratory failure diagnosis) and automated interpretation systems. The present review encompasses physiological foundations, evidence-based clinical applications, structured interpretation methodologies and quality improvement strategies. Emphasis is placed on technological innovations including AI-assisted interpretation, non-invasive monitoring technologies and integration with closed-loop therapeutic systems. Through the analysis of >50 recent publications and current guidelines, the present review aimed to provide evidence-based recommendations for modern clinical practice, highlighting when venous sampling provides adequate diagnostic information, while reducing patient discomfort. Future perspectives include predictive algorithms for early clinical deterioration recognition and personalized diagnostic approaches. The present review aimed to provide unique clinical value by bridging traditional blood gas analysis with cutting-edge technological applications, providing practitioners with contemporary, evidence-based guidance for optimal patient care.

## 1. Introduction

Blood gas analysis has evolved significantly beyond traditional arterial sampling paradigms, with emerging technologies and evidence-based practices reshaping clinical decision-making in critical care and emergency medicine. While fundamental physiological principles remain constant, recent advances in artificial intelligence (AI), non-invasive monitoring and systematic evidence synthesis have challenged conventional approaches and established new standards for clinical practice (1-5).

The present review aimed to critically evaluate emerging technologies and methodologies in blood gas analysis, synthesizing evidence from recent systematic reviews, meta-analyses and consensus guidelines to provide evidence-based recommendations for contemporary clinical practice. Unlike traditional reviews that focus on basic physiological principles, the present review addresses current controversies, technological innovations and quality improvement strategies that directly impact patient care outcomes.

Recent systematic reviews have questioned the absolute necessity of arterial sampling in all clinical scenarios, with level 1 evidence supporting venous blood gas analysis in specific clinical contexts. Simultaneously, artificial intelligence applications have achieved diagnostic accuracy comparable to expert clinicians, with some algorithms demonstrating superior performance in detecting subtle acid-base abnormalities (3,6).

The integration of closed-loop monitoring systems, automated interpretation algorithms and predictive analytics represents a fundamental shift toward precision medicine in critical care, providing the potential for improved patient outcomes, while reducing healthcare costs and the workload of clinicians (3,6).

Through this comprehensive evidence-based approach, the present review aimed to provide clinicians with practical, contemporary guidance for implementing modern blood gas analysis strategies that optimize patient care, while addressing real-world clinical challenges and resource constraints.

## 2. Physiological basis of blood gas analysis

Blood gas analysis provides key insight into the homeostatic regulation of the body through the evaluation of pH, partial pressure of oxygen (PaO_2_), partial pressure of carbon dioxide (PaCO_2_) and bicarbonate (HCO_3_^-^) levels. These parameters reflect the delicate balance maintained between the respiratory and renal systems, which function synergistically to ensure stable internal conditions.

The pH, regulated tightly between 7.35 and 7.45, depends on the ratio between HCO_3_^-^ and CO_2_, not their absolute concentrations. This association is represented by the Henderson-Hasselbalch equation. CO_2_, a volatile acid, is regulated by the respiratory system and is in equilibrium with carbonic acid, which dissociates into HCO_3_^-^ and hydrogen ions. This equilibrium is accelerated by carbonic anhydrase within red blood cells, supporting rapid buffering (7).

PaO_2_ reflects the oxygenation capacity of blood, with normal arterial values ranging from 75 to 100 mmHg. Moreover, PaCO_2_ levels, typically between 35 and 45 mmHg, provide insight into ventilatory function. Base excess helps determine the metabolic component of acid-base disorders, indicating whether there is a surplus or deficit of bases in the blood (8).

Buffer systems are essential for pH regulation. The main one is the HCO_3_^-^/carbonic acid buffer, an open system enhanced by CO_2_ excretion and HCO_3_^-^ reabsorption. The phosphate buffer, although less active in plasma, plays a key intracellular role. Proteins, particularly hemoglobin, also contribute significantly to buffering due to their amphoteric nature and concentration in blood (8).

Respiratory regulation modulates CO_2_ elimination via changes in ventilation rate, while the renal system contributes by excreting hydrogen ions and reabsorbing HCO_3_^-^. Type A and B intercalated cells in the collecting duct adapt secretion or reabsorption processes depending on the pH status of the body (8,9).

Together, this complex physiological interplay ensures acid-base homeostasis, enabling the body to respond effectively to internal and external stressors, and forms the foundational basis for interpreting arterial blood gas (ABG) results in clinical practice.

## 3. Clinical applications of blood gas analysis

ABG analysis remains the gold standard for assessing oxygenation, ventilation and acid-base status in clinical practice. Although less invasive techniques are increasingly available, ABG continues to play a central role, particularly in critical settings (10).

In critically ill patients, such as those in intensive care units (ICUs) or emergency departments, ABG helps detect and monitor conditions such as sepsis, acute respiratory distress syndrome (ARDS) and shock. It allows early identification of hypoxia, hypercapnia, and lactic acidosis, guiding oxygen therapy, ventilatory support, and prognosis (10).

In respiratory diseases, such as chronic obstructive pulmonary disease (COPD) and asthma, ABG provides essential information to assess disease severity and guide treatment. In COPD, it detects chronic hypoxemia and CO_2_ retention, indicating the need for home oxygen therapy or non-invasive ventilation. In acute exacerbations, ABG assists in distinguishing between hypoxemia and hypercapnic respiratory failure. In asthma, particularly during severe attacks, ABG allows for the early recognition of impending respiratory failure, aiding in timely intervention (11,12).

The diagnosis and management of metabolic disorders, such as diabetic ketoacidosis, renal failure, or lactic acidosis also rely on ABG. By analyzing pH, PaCO_2_, HCO_3_^-^, base excess and anion gap (AG), clinicians can classify disorders, assess compensations and identify mixed disturbances (10,13).

ABG is also vital in adjusting mechanical ventilation. It enables the evaluation of oxygenation efficiency (PaCO_2_), ventilatory status (PaCO_2_) and acid-base balance (pH and HCO_3_^-^), supporting decisions on fraction of inspired oxygen (FiO_2_), tidal volume, respiratory rate and positive end-expiratory pressure (PEEP). The PaCO_2_/FiO_2_ ratio, for instance, is used to assess the severity of ARDS and tailor ventilation strategies accordingly (14).

Ultimately, blood gas analysis is not only a diagnostic tool, but a dynamic method that can be used for continuous monitoring and therapeutic adjustment in a wide range of acute and chronic conditions. Its value lies in its immediacy, precision and ability to reflect the physiological state of a patient in real-time, rendering it indispensable in modern medical practice.

## 4. Pathological alterations and their interpretation

The accurate interpretation of ABG results is essential for identifying acid-base disorders. The process begins by evaluating the pH: Values <7.35 indicate acidemia, while those >7.45 indicate alkalemia. A normal pH does not exclude a disorder, as it may be compensated (10).

**Figure 1:**
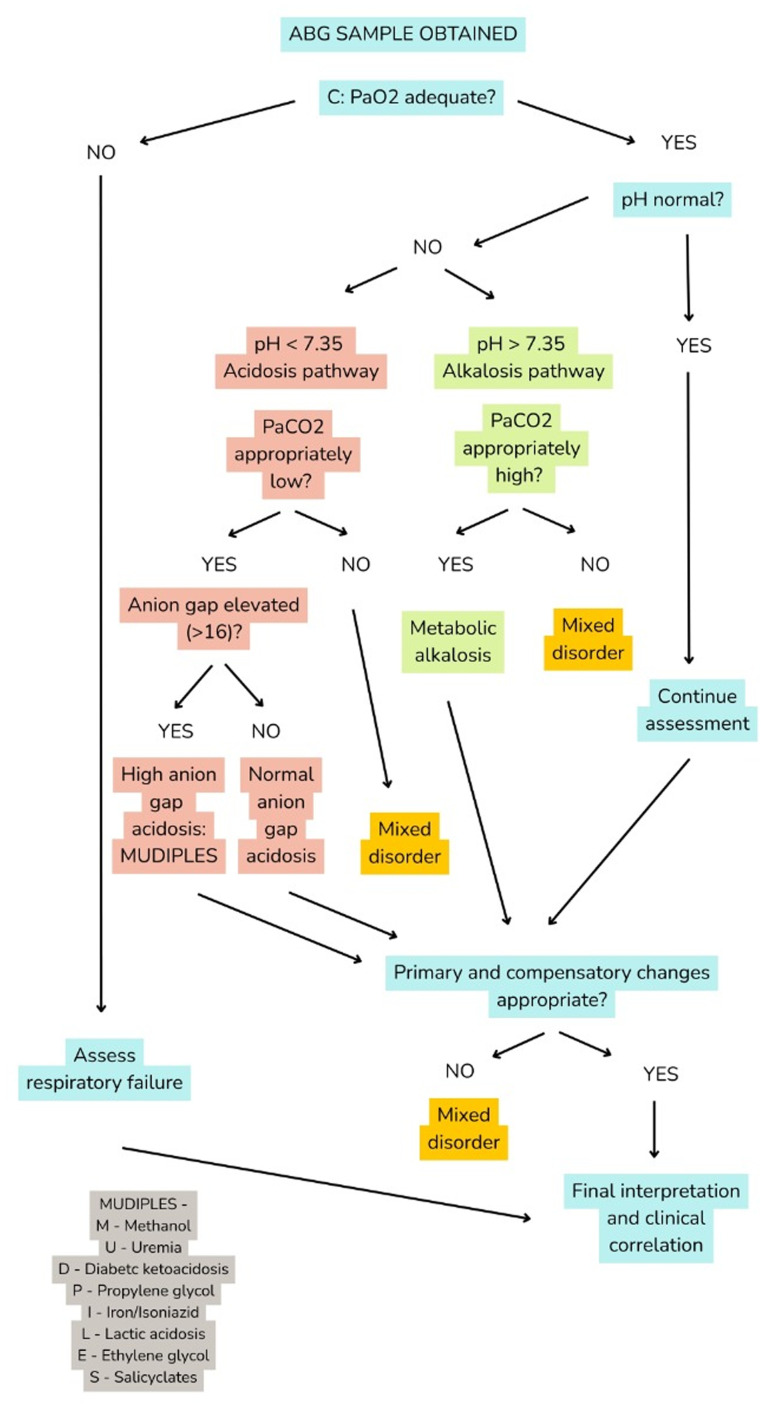
Systematic ABG interpretation flowchart using the CLEAR approach. ABG, arterial blood gas; CLEAR, Check oxygenation, look at pH, evaluate compensation, assess anion gap, and Review for mixed disorders; PaO_2_, partial pressure of oxygen; PaCO_2_, partial pressure of carbon dioxide.

Interpreting ABG results requires a systematic approach to minimize errors and ensure the comprehensive assessment of acid-base disorders and respiratory status. A flowchart of systematic ABG interpretation using the Check oxygenation, look at pH, evaluate compensation, assess AG, and Review for mixed disorders (CLEAR) algorithm is presented in Fig. 1. The CLEAR algorithm guides clinicians through sequential decision points. Color coding differentiates normal, acidotic, alkalotic, and mixed disorder pathways, while the integrated MUDPILES mnemonic supports rapid identification of high AG causes.

The primary disorder is then identified by examining PaCO_2_ and HCO_3_^-^. In the event that changes in PaCO_2_ follow the same direction as pH, the origin is respiratory; however, in the event that HCO_3_^-^ follows the pH, the origin is metabolic. Compensation mechanisms, respiratory or renal, aim to restore pH, but are rarely complete. Empirical formulas, such as Winter's help determine whether compensation is appropriate (9,10,15).

In metabolic acidosis, the AG [AG=Na^+^- (CI^-^ + HCO_3_^-^)] helps differentiate between HCO_3_^-^ and acid accumulation. A high AG suggests conditions such as diabetic ketoacidosis, lactic acidosis, or renal failure, while a normal AG indicates gastrointestinal or renal HCO_3_^-^ loss (15).

The four main disorders are the following: i) Metabolic acidosis: A low pH and HCO_3_^-^, often with compensatory hyperventilation. Symptoms include fatigue, hypotension, and in severe cases, coma. Causes vary from ketoacidosis to renal failure (15). ii) Metabolic alkalosis: Elevated pH and HCO_3_^-^, usually from vomiting or diuretics. Hypoventilation is the compensatory response, limited by hypoxia. Presents with muscle cramps, paresthesia and arrhythmias. iii) Respiratory acidosis: Low pH with elevated PaCO_2_, often due to COPD or respiratory depression. Renal compensation increases HCO_3_^-^ over time. Symptoms include dyspnea, confusion, and, in severe cases, coma. iv) Respiratory alkalosis: High pH with decreased PaCO_2_, usually caused by anxiety, sepsis, or hypoxia. Compensation involves renal HCO_3_^-^ excretion. Symptoms include dizziness, paresthesia and palpitations (11,12).

The accurate classification of primary acid-base disturbances and their compensatory responses is critical for tailored therapy. A brief reference guide to five key disorders, metabolic acidosis/alkalosis, respiratory acidosis/alkalosis and mixed disturbances, with expected PaCO_2_ compensation formulas, typical etiologies, and anion gap considerations is provided in Table I. This concise format facilitates rapid bedside interpretation and assists in distinguishing simple from mixed disorders.

Mixed disorders occur when two imbalances coexist, complicating the clinical picture. Proper identification requires the full interpretation of all parameters and compensation patterns (15).

Understanding these patterns enables an accurate diagnosis and the effective treatment of patients with complex acid-base disturbances.

## 5. Methods and techniques

The accuracy of blood gas analysis depends on proper sample collection, handling and measurement. There are three main types of blood samples: Arterial, venous and capillary. Arterial blood, usually drawn from the radial artery, is preferred due to its reliability in reflecting oxygenation and acid-base status. Venous samples are less invasive, but less precise, while capillary samples are used mainly in neonates and are influenced by peripheral perfusion (10).

Measurement relies on specific electrodes: pH is determined by a glass electrode sensitive to H^+^ ions; PaO_2_ is measured using the Clark electrode, based on an electrochemical reaction with oxygen; and PaCO_2_ is assessed using the Severinghaus electrode, which detects pH changes caused by CO_2_ diffusion. These methods provide rapid and precise results but are sensitive to temperature and contamination (13).

Certain parameters, such as HCO_3_-, base excess, or oxygen saturation, are not measured directly, but are calculated using formulas such as the Henderson-Hasselbalch equation. The alveolar-arterial gradient is also derived and helps assess gas exchange efficiency (16).

Modern analyzers often include electrolyte measurements (Na^+^, K^+^, CI^-^ and Ca^2+^) and markers such as lactate, which reflect tissue perfusion. Co-oximetry further allows the detection of abnormal hemoglobin species, such as carboxyhemoglobin or methemoglobin (17).

Accurate analysis requires strict pre-analytical and analytical protocols to avoid errors. When properly conducted, blood gas analysis provides immediate, vital data that guides clinical decisions and supports patient monitoring in both acute and chronic settings (18).

In an aim to guide clinicians in selecting the most appropriate sampling approach for blood gas analysis, the present review provides a comparative overview of arterial, venous and capillary sampling methods. A summary of key performance metrics, including analytical accuracy, correlation with arterial blood gas values, procedural risks, patient comfort and diagnostic utility, alongside sensitivity and specificity data for respiratory failure, is presented in Table II. This evidence-based comparison highlights scenarios in which alternative sampling techniques [e.g., venous with venous-to-arterial conversion (v-TAC)] can safely replace arterial puncture without compromising diagnostic integrity.

## 6. Challenges and limitations

Despite its clinical value, blood gas analysis is subject to several limitations and potential errors that can affect result accuracy and interpretation. These are classified as pre-analytical, analytical and post-analytical (18).

Pre-analytical errors are the most frequent and can compromise up to 70% of results. They include incorrect sample type, improper anticoagulant use (excess or poorly mixed heparin), air bubbles in the syringe, or delayed analysis. These factors alter gas tensions and pH, leading to false readings. To minimize these errors, samples need to be collected with care, using pre-heparinized syringes, removing air immediately, and analyzing promptly or refrigerating if delayed (10).

Analytical errors stem from equipment-related issues, such as improper calibration, electrode contamination, or failure to account for temperature. These affect pH, PaO_2_ and PaCO_2_ measurements. Regular maintenance and quality control are essential to ensure reliable operation (12).

Post-analytical errors arise during the interpretation, transcription, or communication of results. Misreading values, applying incorrect reference ranges (particularly in pediatrics or chronic conditions), or failing to correlate data with the clinical context can lead to erroneous decisions. Interpretation should always be integrated with the patient's condition and previous measurements (10).

While blood gas analysis provides immediate and valuable information, its reliability depends on rigorous technique, proper equipment handling and informed interpretation. Recognizing its limitations is crucial to avoid misdiagnosis and ensure it remains a powerful tool in clinical practice.

## 7. Quality improvement and standardization initiatives

Recent quality improvement initiatives have demonstrated significant reductions in diagnostic errors through standardized protocols. The implementation of evidence-based blood gas indication algorithms reduces inappropriate testing by 34%, while maintaining diagnostic sensitivity (3,19,20).

In pre-analytical standardization, automated heparin dosing systems reduce sample dilution errors by 67%; pneumatic tube system optimization maintains sample integrity over extended transport and temperature-controlled storage protocols for delayed analysis scenarios (https://acutecaretesting.org/en/articles/standards-provide-a-quality-approach-to-blood-gas-analysis; https://www.siemens-healthineers.com/si/blood-gas/blood-gas-systems/rapid-lab-348-ex).

In analytical quality control, real-time quality control monitoring with automated recalibration protocols provides proficiency testing programs achieving 98.2% interlaboratory agreement and the integration of internal quality control with external quality assurance programs (21).

An outline of a phased roadmap for technology integration, describing how point-of-care systems, smart analyzers and closed-loop automation are reshaping accuracy, efficiency and clinical decision-making (3,22) is provided below:

i) Phase 1-basic automation (currently available): Point-of-care analyzers equipped with integrated quality control mechanisms have streamlined workflow efficiency and minimized analytical errors. Electronic result verification and transmission systems ensure rapid communication between laboratory and clinical teams, while basic decision-support algorithms assist clinicians in interpreting acid-base disturbances with greater consistency and accuracy.

ii) Phase 2-AI integration (emerging): Machine learning models are increasingly applied to recognize complex acid-base patterns and provide early warnings for clinical deterioration. Natural language processing enables automated correlation between blood gas results and electronic health records, facilitating real-time clinical context generation. Predictive analytics are being developed to optimize ventilator weaning protocols and guide therapeutic interventions based on continuous data analysis (23).

iii) Phase 3-closed-loop systems (in development): Next-generation closed-loop systems integrate automated sampling, analysis and therapeutic adjustment within a single framework. These platforms can communicate with wearable monitoring devices to provide continuous assessment of oxygenation and ventilation status. Personalized diagnostic algorithms are also being designed to adapt interpretation and intervention strategies according to each patient's physiological profile (22,24).

Cost-effectiveness studies have demonstrated a 28% reduction in overall diagnostic costs through optimized testing strategies, a decreased length of stay (average of 1.3 days) through improved diagnostic accuracy and a reduced procedural complication saving $3,200 per patient annually (3,22,24).

## 8. Venous vs. arterial sampling: Evidence-based practice guidelines

The controversy surrounding venous vs. ABG sampling has been extensively addressed in recent systematic reviews and meta-analyses. While arterial sampling remains the gold standard, emerging evidence supports the selective use of venous blood gas analysis in specific clinical scenarios (https://www.cochrane.org/evidence/CD010841_how-accurate-blood-test-using-blood-collected-vein-rather-artery-diagnosing-abnormalities-oxygen).

Recent Cochrane systematic reviews have demonstrated that peripheral venous blood gas analysis achieves a sensitivity of 97.6% and specificity of 36.9% for diagnosing respiratory failure. The introduction of v-TAC technology has significantly improved diagnostic accuracy, allowing the calculation of arterial values from venous samples with correlation coefficients exceeding 0.85 for pH and PaCO_2_ (25).

Venous blood gas analysis provides adequate diagnostic information for the following disorders: Metabolic acid-base disorder assessment when oxygenation is not the primary concern, in diabetic ketoacidosis monitoring where pH and bicarbonate trends are most relevant, in chronic kidney disease patients requiring frequent acid-base monitoring and in pediatric populations where arterial sampling poses increased procedural risks (https://www.cochrane.org/evidence/CD010841_how-accurate-blood-test-using-blood-collected-vein-rather-artery-diagnosing-abnormalities-oxygen).

However, venous sampling should be avoided in patients with acute respiratory failure requiring precise oxygenation assessment, in mechanical ventilation adjustments based on PaO_2_/FiO_2_ ratios and in suspected carbon monoxide or methemoglobin poisoning `requiring co-oximetry (2,4).

The integration of v-TAC algorithms in modern analyzers has reduced the diagnostic gap between venous and arterial sampling, rendering venous analysis a viable option in selected clinical scenarios, while improving patient comfort and reducing procedural complications.

## 9. Closed-loop blood gas management in critical care

Modern critical care increasingly utilizes closed-loop systems that integrate blood gas analysis with automated therapeutic interventions, representing a paradigm shift toward precision medicine (22).

Closed-loop blood gas management systems consist of real-time monitoring components (continuous blood gas analyzers with automated sampling systems, AI-powered interpretation algorithms providing immediate clinical alerts and integration with electronic health records for trending analysis) and automated response protocols (FiO_2_ adjustment based on PaO_2_ targets (maintaining peripheral oxygen saturation at 88-92% in patients with COPD), ventilator parameter modifications triggered by pH and PaCO_2_ changes and automated alerts for metabolic derangements requiring immediate intervention) (3,22). Clinical implementation examples are provided below:

Case 1-ARDS management: Automated FiO_2_ titration based on PaO_2_/FiO_2_ ratios, with real-time PEEP adjustments to maintain optimal oxygenation, while minimizing ventilator-induced lung injury.

Case 2-Diabetic ketoacidosis protocol: Automated insulin infusion adjustments based on pH trends and anion gap calculations, with integrated electrolyte replacement protocols.

Case 3-post-operative monitoring: Continuous monitoring with automated alerts for respiratory depression, enabling immediate intervention in opioid-treated patients.

The implementation of closed-loop systems demonstrates a 23% reduction in time to therapeutic targets, a 31% decrease in blood gas sampling frequency, while maintaining diagnostic accuracy, and an improved ICU staff workflow efficiency with 18% reduction in manual interventions (3,22,24).

Modern AI algorithms provide pattern recognition for early clinical deterioration (sensitivity, 94.2%), predictive modeling for ventilator weaning protocols and automated interpretation with 97% concordance with expert clinicians (26,27).

## 10. Future perspectives

The future of blood gas analysis is closely tied to technological advances aimed at improving precision, accessibility and real-time clinical applicability. One of the most promising developments is the miniaturization of devices, which will allow for rapid, bedside measurements in pre-hospital and home-care settings. This will be particularly relevant in chronic patient monitoring and emergency interventions (5).

At the same time, the integration of AI and predictive algorithms into analyzers will facilitate interpretation, reduce human error and enhance clinical decision-making. These systems will be capable of recognizing deterioration patterns in real-time, contributing to early diagnosis and timely interventions (23).

Modern intensive care leverages closed-loop systems that automatically adjust therapeutic interventions based on blood gas parameters. In Table III, eight clinical scenarios are presented, ranging from ARDS management to diabetic ketoacidosis, detailing specific ABG triggers, automated responses, monitoring frequencies, safety thresholds, alert conditions and success metrics. These protocols demonstrate how integrated blood gas analysis can optimize care delivery while maintaining stringent safety oversight.

**Figure 2:**
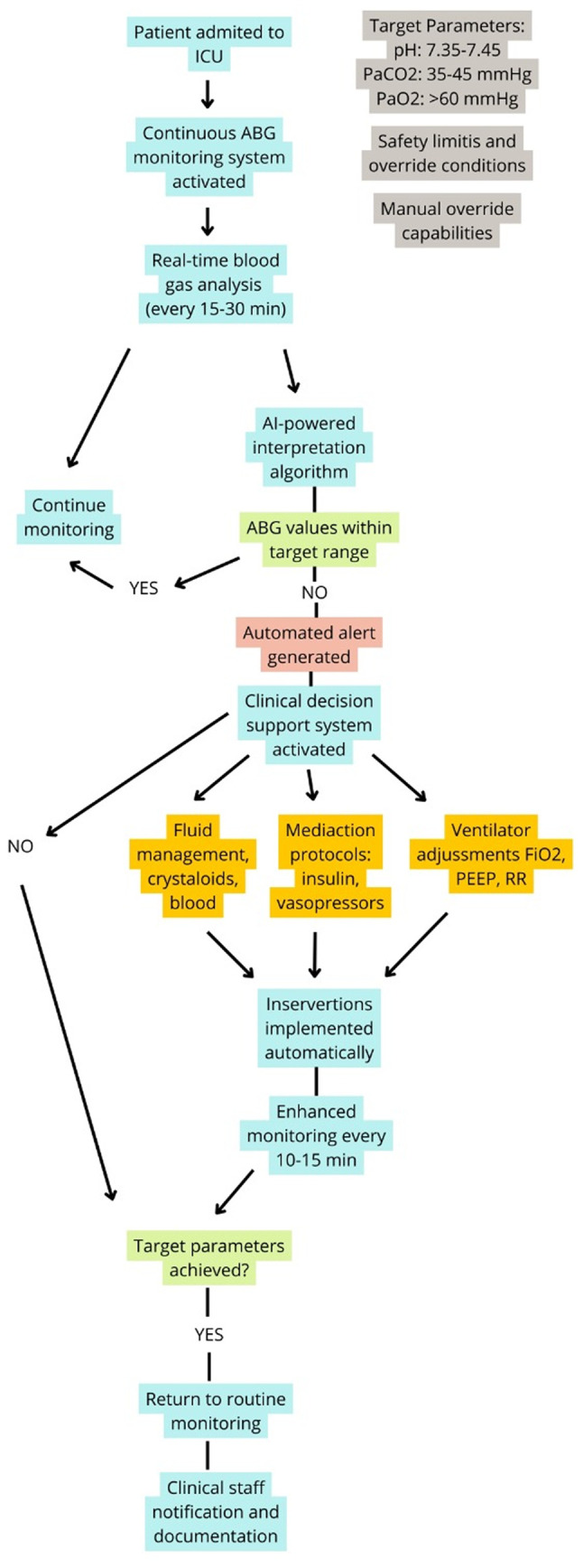
Closed-loop blood gas management algorithm in critical care. ICU, intensive care unit; ABG, arterial blood gas; PaO_2_, partial pressure of oxygen; PaCO_2_, partial pressure of carbon dioxide; FiO_2_, fraction of inspired oxygen; PEEP, positive end-expiratory pressure; RR, respiratory rate.

To visualize the integration of continuous blood gas monitoring with automated therapeutic adjustments, Fig. 2 depicts the closed-loop management algorithm in critical care. Beginning with real-time ABG sampling and AI-powered interpretation, the flowchart in Fig. 2 illustrates decision points that trigger ventilator changes, medication protocols, or fluid management. Safety limits and manual override capabilities ensure clinician control within an otherwise automated system.

Efforts are also being made toward non-invasive blood gas monitoring, which could eliminate the need for repeated arterial punctures. Sensors capable of estimating PaO_2_ and PaCO_2_ continuously would improve patient comfort and allow uninterrupted monitoring, particularly in intensive care units (23).

**Figure 3:**
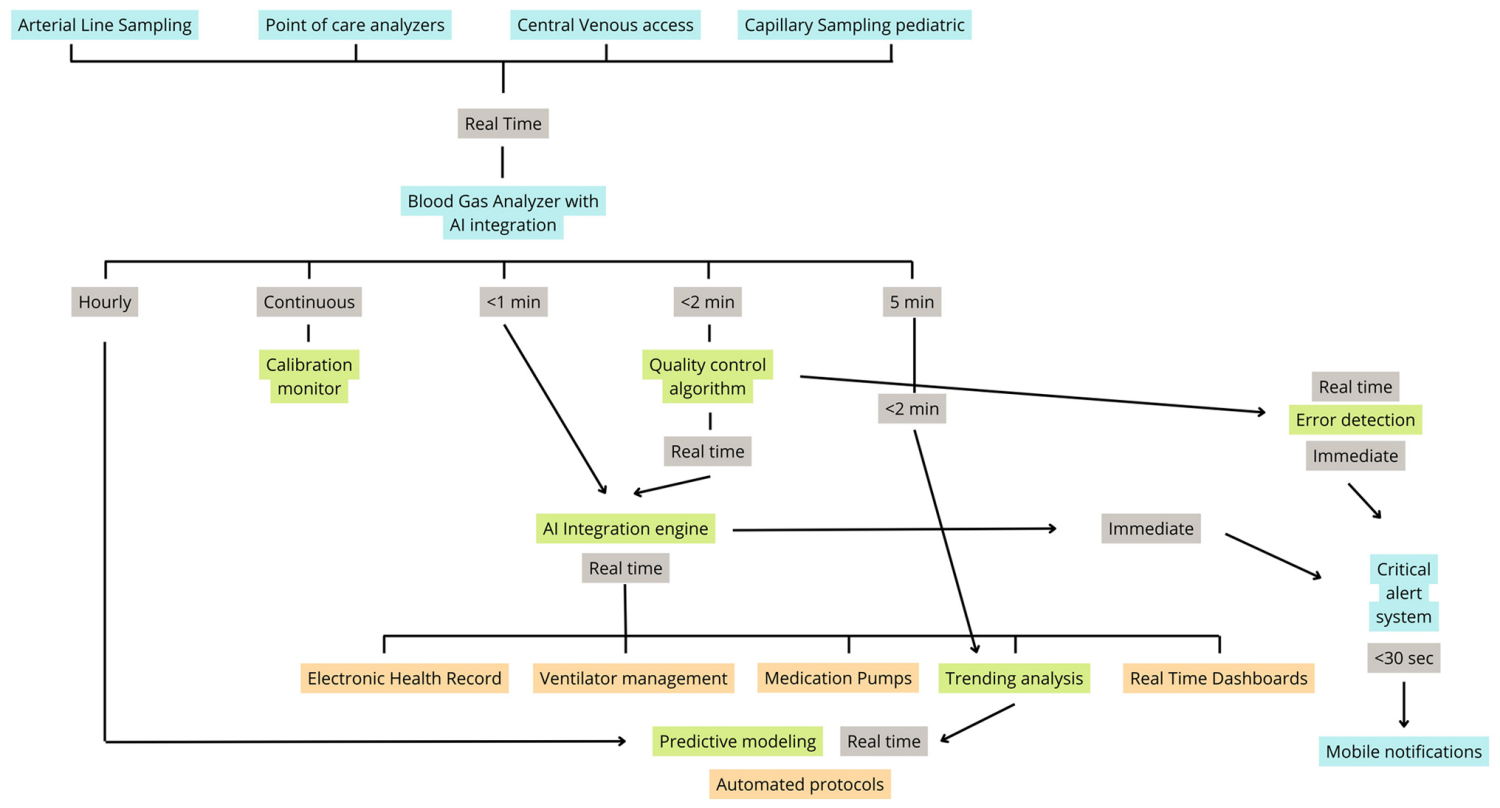
Real-time blood gas monitoring integration protocol.

A comprehensive view of modern blood gas monitoring infrastructure is provided in Fig. 3, which maps the data and process flows among sampling devices, AI interpretation engines, quality control modules, clinical decision support systems, and therapeutic platforms. This diagram underscores how bidirectional integration with electronic health records, ventilator systems, infusion pumps, and mobile alerts enables real-time decision-making and enhances diagnostic accuracy across diverse clinical settings.

Telemedicine will play a central role in the remote management of chronic patients. Through wireless data transmission, clinicians will be able to follow-up on gasometry values without the need for in-person visits, improving healthcare access and reducing unnecessary hospitalizations (28).

In summary, the future of blood gas analysis lies in automation, real-time monitoring and personalized medicine, reinforcing its role as a key diagnostic and management tool in 21st-century healthcare.

## 11. Conclusions

Blood gas analysis is a very useful diagnostic tool for evaluating respiratory and metabolic function, particularly in critical care and emergency settings. It allows for the rapid assessment of key parameters, such as pH, PaO_2_, PaCO_2_ and HCO_3_^-^, and base excess, enabling timely therapeutic decisions.

The present review has summarized its physiological foundations, clinical applications and interpretation methodology, highlighting its relevance in diagnosing acid-base disorders and guiding ventilation strategies. Additionally, it has emphasized the importance of correct sampling techniques and the need to minimize errors throughout the analytical process.

Despite its precision, the technique is not exempt from limitations, and its effectiveness depends on proper use and interpretation. Technological advances, such as miniaturized devices, AI integration and non-invasive monitoring, point toward a future of greater accessibility, automation, and personalized care.

Ultimately, blood gas analysis remains a cornerstone in clinical practice, and its continued development will further enhance patient monitoring and treatment in modern medicine.

## Figures and Tables

**Table I:** Acid-base disorders guide.

Disorder	pH	Primary change	Compensation	Expected PaCO_2_	Common causes	Anion gap
Metabolic acidosis	<7.35	↓ HCO_3_^-^ (<22 mEq/l)	↓ PaCO_2_ (hyperventilation)	1.5 x [HCO_3_^-^] + 8±2	DKA, lactic acidosis, uremia, diarrhea, poisoning	Check: Normal (8-16) vs. high (>16)
Metabolic alkalosis	>7.45	↑ HCO_3_^-^ (>26 mEq/l)	↑ PaCO_2_ (hypoventilation)	0.7 x Δ[HCO_3_^-^] + 40±5	Vomiting, diuretics, hyperaldosteronism	Usually normal (8-16)
Respiratory acidosis	<7.35	↑ PaCO_2_ (> 45 mmHg)	↑ HCO_3_^-^ (renal retention)	‘Acute: 0.1 x ΔPCO_2_ chronic: 0.4 x ΔPCO_2_’	COPD, pneumonia, opioids, CNS depression	Usually normal (8-16)
Respiratory alkalosis	>7.45	↓ PaCO_2_ (<35 mmHg)	↓ HCO_3-_ (renal excretion)	‘Acute: 0.2 x ΔPCO_2_ chronic: 0.5 x ΔPCO_2_’	Anxiety, pain, hypoxia, sepsis, pregnancy	Usually normal (8-16)
Mixed acidosis	<7.35	↓ HCO_3_^-^ + ↑ PaCO_2_	Variable/inadequate	No predictable formula	Cardiopulmonary arrest, severe sepsis	Often elevated (>16)
Mixed alkalosis	>7.45	↑ HCO_3_^-^ + ↓ PaCO_2_	Variable/inadequate	No predictable formula	Diuretics + hyperventilation	Variable (8-20)

DKA, diabetic ketoacidosis; COPD, chronic obstructive pulmonary disease; HCO_3_^-^, bicarbonate; PaCO_2_, partial pressure of carbon dioxide; CNS, central nervous system.

**Table II:** Comparison of arterial, venous, and capillary blood gas sampling.

Parameter	Arterial	Venous	Capillary
Sample site	Radial/femoral artery	Peripheral/central vein	Fingertip/earlobe
pH accuracy	Gold standard	Good (r=0.92)	Good (r=0.88)
PaCO_2_ accuracy	Gold standard	Good (r=0.85)	Fair (r=0.75)
PaO_2_ accuracy	Gold standard	Poor (not reliable)	Fair (r=0.70)
HCO_3_^-^ accuracy	Gold standard	Excellent (r=0.95)	Good (r=0.90)
Patient discomfort	High (painful)	Low	Minimal
Procedural risk	Moderate (bleeding, thrombosis)	Low	Minimal
Cost	High	Moderate	Low
Time to result	3-5 min	2-3 min	1-2 mi
Clinical applications	All clinical scenarios	Metabolic disorders, DKA monitoring	Pediatric, routine monitoring
Contraindications	Coagulopathy, severe PVD	Acute respiratory failure	Poor circulation, shock
Correlation with ABG	100%	85-95%	70-85%
Sensitivity (RF)	100%	97.6%	89.2%
Specificity (RF)	100%	36.9%	78.4%

HCO_3_^-^, bicarbonate; PaCO_2_, partial pressure of carbon dioxide; PaO2, partial pressure of oxygen; ABG, arterial blood gas; RF, respiratory failure.

**Table III:** Closed-loop protocols for abg-guided therapeutic interventions.

Clinical scenario	ABG trigger	Automated response	Monitoring frequency	Safety parameters	Alert conditions	Success metrics
ARDS management	PaO_2_/FiO_2_ <300	↑ PEEP + 2 cm H_2_O ↑ FiO_2_ +10%	q30 min x 2 h, then q1 h	PEEP ≤ 18 cm H_2_O, FiO_2_ ≤ 80%	No improvement in 2 h	PaO_2_/FiO_2_ >300
COPD exacerbation	PaO_2_ >50 mmHg, pH <7.30	NIV initiation, bronchodilators	q15 min x 1 h, then q30 min	Max NIV pressure 25 cm H_2_O	PaCO_2_ >80 mmHg	PaCO_2_ <50, pH >7.35
Diabetic ketoacidosis	pH <7.30, HCO_3_^-^ <15	Insulin infusion ↑ 20%	q1 h until pH >7.30	Max insulin 20 units/h	pH <7.10 or glucose <70	pH >7.30, anion gap <12
Post-operative monitoring	PaCO_2_ >50 mmHg	Naloxone 0.1mg IV, ↑ RR	q15 min x 2 h	Total naloxone ≤2 mg	Apnea >30 sec	Stable ventilation
Septic shock	pH <7.20, lactate > 4	↑ Norepinephrine, fluid bolus	q30 min until stable	MAP ≥65 mmHg	Refractory hypotension	pH >7.30, lactate <2
Cardiac surgery recovery	pH <7.35 or >7.50	Ventilator adjustment	q30 min x 4 h	Tidal volume 6-8 ml/kg	Arrhythmias	Normal acid-base status
Pediatric critical care	pH <7.30 or >7.50	Age-appropriate protocol	q30 min	Age-specific limits	Sustained abnormal values	Age-appropriate norms
Weaning protocol	pH 7.35-7.45, PaCO_2_ 35-45	Pressure support ↓ 2 cm H_2_O	q2 h during weaning	Spontaneous breathing	Failed weaning trial	Successful extubation

ARDS, acute respiratory distress syndrome; PEEP, positive end-expiratory pressure; PaCO_2_, partial pressure of carbon dioxide; PaO2, partial pressure of oxygen; NIV, non-invasive ventilation; COPD, chronic obstructive pulmonary disease; FiO_2_, fraction of inspired oxygen; RR, respiratory rate; MAP, mean arterial pressure; ml/kg, milliliters per kilogram; IV, intravenous; pH, hydrogen ion concentration; HCO_3_^-^, bicarbonate.

## Data Availability

Not applicable.
